# Re-bleeding and its predictors after capsule endoscopy in patients with obscure gastrointestinal bleeding in long-term follow-up

**DOI:** 10.1186/s12876-019-1137-3

**Published:** 2019-12-16

**Authors:** Panu Wetwittayakhlang, Jirapat Wonglhow, Nisa Netinatsunton, Naichaya Chamroonkul, Teerha Piratvisuth

**Affiliations:** Gastroenterology and Hepatology Unit, Division of Internal Medicine, Faculty of Medicine, Prince of Songkla University, Songkhla, 90110 Thailand

**Keywords:** Capsule endoscopy, Obscure gastrointestinal bleeding, Long-term, Re-bleeding, Predictors

## Abstract

**Background:**

Capsule endoscopy (CE) is the preferred diagnostic test of choice in the investigation of obscure gastrointestinal bleeding (OGIB). Although, a conservative strategy is recommended in the short-term, for cases with a negative result from CE, the impact of CE on long-term re-bleeding still remains unclear. Hence, the aim of this study was to determine the long-term re-bleeding rate along with predictors after CE in patients with OGIB.

**Methods:**

We retrospectively reviewed 216 patients with OGIB, whom had received a CE examination, so as to investigate the cause of obscure GI bleeding; between July 2008 and March 2018. The patient’s characteristics, medication use, CE finding, treatments strategy, re-bleeding episodes and follow-up information were collected from the institutional electronic medical chart and CE database. Re-bleeding free survival was evaluated using Kaplan-Meier curves with log rank test, whilst predictors associated with the re-bleeding episodes were analyzed via the use of Cox proportional hazard model.

**Results:**

One hundred and thirty-three patients with OGIB, having received CE were enrolled in the analysis. The pool rate of re-bleeding was 26.3% (35/133) during a follow-up duration of 26 months after CE. Patients with positive CE study, without specific treatment, had higher rates of re-bleeding (47.6%) than those with positive study whom received specific treatment (25.7%), and negative study (20.8%) (*p* = 0.042). Although, the re-bleeding free survival was not significantly different among the groups (log rank test; *P* = 0.10). Re-bleeding events occurring within 6, 12, and 24 months after CE were 36, 64 and 92%, respectively. The high-frequency re-bleeding etiologies were the small bowel angiodysplasias and abnormal vascular lesions. Furthermore, independent predictors for re-bleeding after CE were patients with cirrhosis (hazard ratio, HR 4.06), incomplete CE visualization (HR 2.97), and a history of previous GI bleeding (HR 2.80).

**Conclusions:**

The likelihood of re-bleeding after CE was higher in patients with positive CE study than those with negative study. Specific treatments, or therapeutic interventions for patients with detectable lesions reduced the probability of re-bleeding episodes in long-term follow-up. Close follow-up for recurrent bleeding is recommeded for at least 2 years after CE.

## Background

Obscure gastrointestinal bleeding (OGIB) is defined as recurrent or persistent gastrointestinal (GI) bleeding from a source that remains unidentified after esophagogastroduodenoscopy (EGD) and colonoscopy. OGIB is classified into 2 types: overt and occult. Overt bleeding is characterized by presence of visible blood (melena or hematochezia), whilst occult bleeding is characterized by iron-deficiency anemia, or positive fecal occult blood [1, 2]. In approximately 75% of OGIB patients, bleeding lesions can be detected in the small bowel [2].

Capsule endoscopy (CE) is the diagnostic test of choice for investigation of OGIB. The diagnostic yield of CE is comparable with device-assisted enteroscopy (DAE) [3, 4]. Recent guidelines of the European Society of Gastrointestinal Endoscopy endorse CE as the first-line small bowel investigation for patients with OGIB. When a lesion is detected certain, specific therapies (endoscopic, surgical, or medical treatment) may be performed, while in the event of a negative CE result, a “watch and wait strategy” is recommended in the case of no on-going bleeding [5]. Since previous studies [6, 7] showed a good prognosis and low risk of re-bleeding, a conservative approach is favored in the short-term. However, this strategy is not universally accepted because, data on the re-bleeding rates among patients in the long-term with a negative CE showed results that varied from 4.8 to 36% [7–14]. Undoubtedly, the limitations of CE included missing significant lesions and the inability to provide therapy.

Although, previous studies from eastern countries [7, 8, 15–17] revealed that the possibility of re-bleeding is still high, despite negative result of CE, few studies have provided long-term follow-up from eastern countries. Furthermore, the data were inconsistent among the study populations, and there were different etiologies of the bleeding lesions. The aim of this study was to determine the recurrent bleeding rate after CE in the long-term, so as to identify the risk factors associated with re-bleeding episodes in obscure GI bleeding patients.

## Methods

### Patients and study design

A total of 216, consecutive patients with OGIB, whom underwent CE to investigate the cause of obscure GI bleeding in the NKC Institute of Gastroenterology and Hepatology at Songklanagarind Hospital, Songkhla, Thailand, between; July 2008 and March 2018. All patients required both diagnostic EGD and colonoscopy examinations, which results were considered inadequate to explain the patient’s clinical conditions prior to CE. OGIB was classified into two presentations: obscure-overt GI bleeding, which presented with recurrent passage of visible blood (hematochezia or melena); obscure-occult GI bleeding, which presented with positive fecal occult blood test, and/or recurrent iron-deficiency anemia from chronic gastrointestinal blood loss. Patient’s data and follow-up information were collected from the hospital electronic medical chart and CE database. The variable data included age, gender, co-morbidity, medication use (anticoagulant, antiplatelets, and non-steroidal anti-inflammatory drugs, NSAIDs), presentation of OGIB (overt or occult), history of previous overt GI bleeding, hemoglobin level, CE finding, type of specific treatment and time interval to re-bleeding event after CE. The study was reviewed and approved by the Human Research Ethics Committee of the Faculty of Medicine, Prince of Songkla University.

### CE procedure and diagnostic evaluation

For patients with OGIB, CE examinations were conducted in ambulatory patients and hospital inpatients, using PillCam™ SB (Given Imaging, Yoqneam, Israel). Protocol for bowel preparation was applied for all patients, using 2 l of oral polyethylene glycol solution, 6 h before the CE examination. The capsule was swallowed and the images were recorded using a wireless data recorder, for 8–12 h. The recorded CE images were independently reviewed, and evaluated by four, experienced board-certified gastroenterologists.

According to practice guidelines, purposed by Saurin et al. study [5], the small bowel lesions detected by the CE were classified into three types: (I) lesions with a high potential for bleeding (P2); (II) lesions considered to have uncertain bleeding potential (P1); and (III) lesions with no bleeding potential (P0). Positive CE studies were defined as: studies that identified at least one of the P2 lesions, which explained the patient’s clinical conditions (For example active bleeding, angiodysplasias, tumor or ulcer), whereas lesions that were identified with only having P1 or P0 were interpreted as negative CE studies (normal, small erosion and minimal mucosal change).

### Therapeutic strategy after CE

Therapeutic management after CE depended on each patient’s CE result. The treatment was classified into two groups. The first group included patients with identifiable causes on CE. This group received specific treatments of either invasive therapy (therapeutic DAE, embolization, or surgery), or appropriate medical treatment that included muco-protective agents, such as rebamipide and antiangiogenic agents. Types of the therapeutic interventions were chosen based on etiology of the bleeding, and the patient’s condition. The second group received non-specific treatments, such as blood transfusions, iron supplementation and clinical follow-up; if the CE findings were non-significant lesions.

### Follow-up and outcome measurements

Patient information was obtained from the CE database and the electronic medical records, which included recurrence of bleeding after CE, date of re-bleeding, hemoglobin levels and the use of medications, such as antiplatelet use, anticoagulant use, and NSAIDs use. Duration of follow-up has been defined as: the period between the CE study, and the date of re-bleeding, or the last follow-up visit. We excluded patients with a follow-up time of less than 12 weeks, or those with insufficient of data to evaluate the re-bleeding event during the follow-up.

The primary outcome of this study was recurrent bleeding after CE. Re-bleeding defined as: recurrent bleeding episodes as overt bleeding defined by the presence of melena or hematochezia with a drop in hemoglobin > 2 g/dL, or occult bleeding defined as: an unexplained hemoglobin drop of more than 2 g/dL in the absence of melena or hematochezia. Secondary outcomes were the predictors of re-bleeding after CE.

### Statistical analysis

Continuous variables are expressed as mean ± standard deviation (SD), or median and percentiles (interquartile range [IQR] 25th–75th percentile). These were then compared using the t-test or ANOVA test. Categorical variables are expressed as number and percentages, with a 95% confidence interval (CI), and compared by the χ^2^ test or Fisher’s exact test. A Kaplan-Meier curve with the log-rank test was used to evaluate the long-term outcome of re-bleeding survival rate. The re-bleeding variable was defined as the event. The predictors for re-bleeding were analyzed by univariate analysis, using the Cox proportional hazards regression model. In the multivariate analysis, we included only the variables with a *P* value of less than 0.2, from the univariate analysis. A *P* value < 0.05 was considered statistically significant. All analyses were performed using R Studio (version 1.0.153).

## Results

**Fig. 1:**
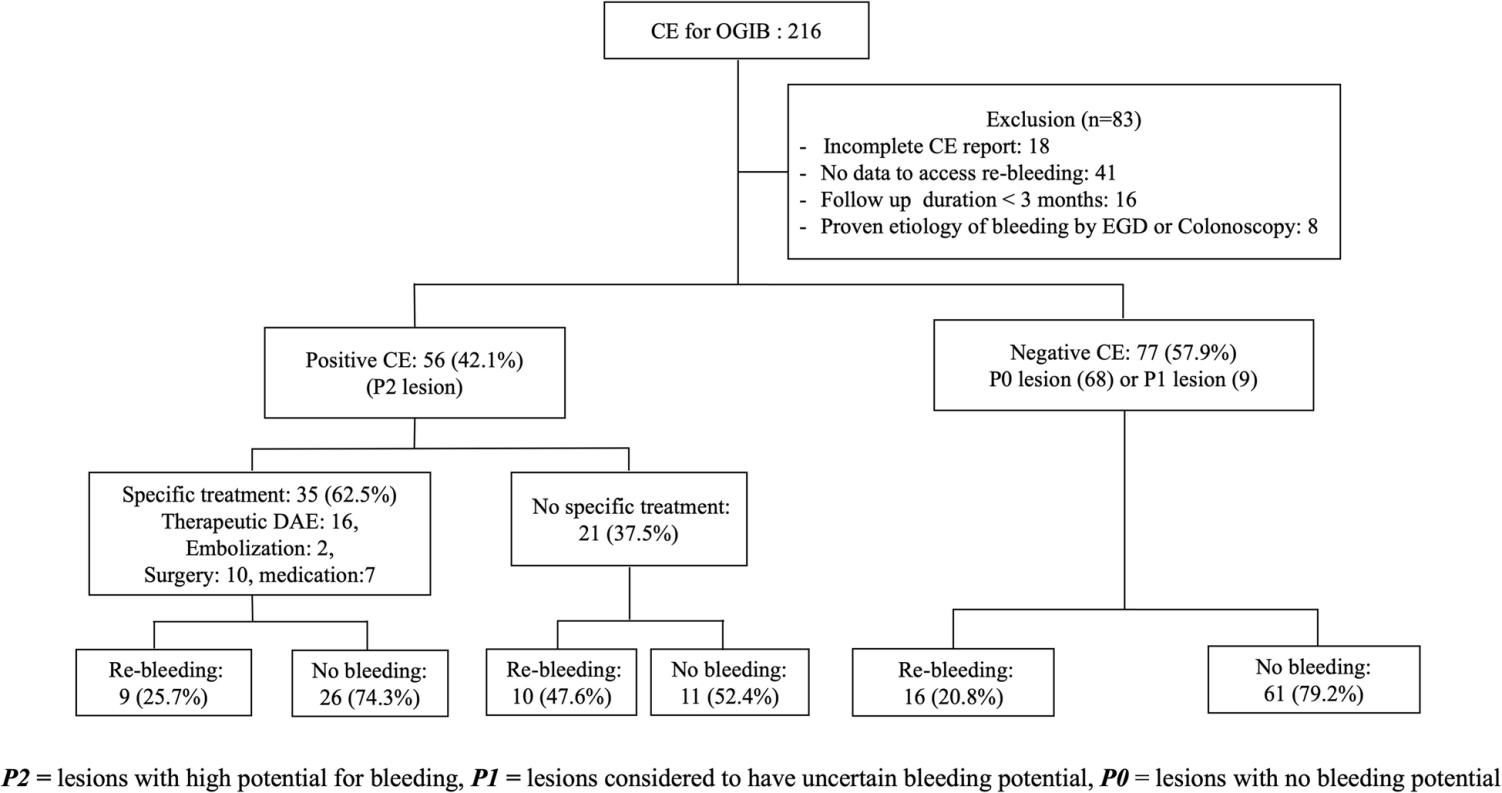
Clinical course of 133 capsule enteroscopy study subjects for obscure GI bleeding. *OGIB* obscure gastrointestinal bleeding, *CE* capsule endoscopy, *DAE* (double balloon endoscopy), *EGD* esophagogastroduodenoscopy

A flow chart of the study is shown in Fig. 1. Overall, 216 patients underwent CE for OGIB. Eighty-three patients were excluded: 18 had incomplete reports of CE, 41 patients had insufficient information to evaluate the re-bleeding event, 16 patients had a follow-up duration of less than 3 months, and 8 patients had a proven etiology of bleeding by EGD or colonoscopy. In total 133 patients were included in the analysis.

### Patient characteristics

The characteristics of the patients are shown in Table 1. The mean ± SD age was 65.4 ± 13.8 years; 51.9% were female. Fifty-eight patients (43.6%) had a history of previous overt GI bleeding before they underwent CE. The common comorbidities were hypertension (48.9%), diabetes mellitus (28.6%) and ischemic heart disease (26.3%). There were no significant differences in co-morbidity, or underlying diseases among the groups. Patients who had positive CE, with specific treatment had a higher percentage of requiring a blood transfusion than the others. The use of aspirin, dual antiplatelet therapy, warfarin and NSAIDs were not different among the groups. The time intervals of performing CE after the onset of a bleeding event was extended in patients with negative CE, more so than those patients with a positive CE result (16 versus 9 days, *P* = 0.025). The CE achieved complete small bowel visualization in most of the patients (91.7%), without any differences between the positive and negative CE groups.

**Table 1:** Clinical characteristics of the study subjects classified by CE results and specific treatments

Characteristics	Overall	Positive CE		Negative CE (*N* = 77)	*P*-value
		Without treatment (*N* = 21)	With treatment (*N* = 35)		
Male	64 (48.1)	11 (52.4)	14 (40.0)	39 (50.6)	0.529
Age in years, mean (SD)	65.4 (13.8)	66.8 (17)	68.5 (15.5)	63.9 (13.3)	0.388
Co-morbidity and underlying diseases					
History of previous overt GI bleeding	58 (43.6)	12 (57.1)	17 (48.6)	29 (37.7)	0.221
Ischemic heart disease	35 (26.3)	7 (33.3)	10 (28.6)	18 (23.4)	0.616
Aortic stenosis	6 (4.5)	0 (0)	3 (8.6)	3 (3.9)	0.425
Hypertension	65 (48.9)	11 (52.4)	19 (54.3)	35 (45.5)	0.646
Diabetes mellitus	38 (28.6)	4 (19.0)	11 (31.4)	23 (29.9)	0.566
Cirrhosis	18 (13.5)	5 (23.8)	4 (11.4)	9 (11.7)	0.331
Chronic hepatitis (HBV, HCV)	8 (6)	1 (4.8)	2 (5.7)	5 (6.5)	1.000
Chronic kidney disease	26 (19.5)	1 (4.8)	11 (31.4)	14 (18.2)	0.056
Thrombocytopenia	8 (6.0)	2 (9.5)	1 (2.9)	5 (6.5)	0.604
Mode of bleeding (Overt)	81 (60.9)	15 (71.4)	26 (74.3)	40 (51.9)	0.045
Melena	41 (30.8)	8 (38.1)	14 (40.0)	19 (24.7)	0.195
Hematochezia	40 (30.1)	7 (33.3)	12 (34.3)	21 (27.3)	0.709
Mode of bleeding (Occult)	52 (39.1)	6 (28.6)	9 (25.7)	37 (48.1)	0.045
Hemodynamic instability	16 (12.0)	5 (23.8)	3 (8.6)	8 (10.4)	0.232
Medications used					
Aspirin	17 (12.8)	3 (14.3)	3 (8.6)	11 (14.3)	0.761
Dual antiplatelet therapy	14 (10.5)	1 (4.8)	5 (14.3)	8 (10.4)	0.577
NSAIDs	15 (11.3)	2 (9.5)	3 (8.6)	10 (13.0)	0.927
Anticoagulants	7 (5.3)	2 (9.5)	3 (8.6)	2 (2.6)	0.207
Lowest Hb level at initial bleeding (g/dL), mean (SD)	7.3 (2.0)	7.2 (2.3)	6.5 (1.7)	7.7 (1.9)	0.018
Drop in Hb level, mean (SD)	3.7 (1.8)	4 (1.7)	4 (1.7)	3.4 (1.8)	0.214
Blood transfusion ≥3 units	57 (42.9)	8 (38.1)	24 (68.6)	25 (32.5)	0.001
Time to CE after bleeding in day, median (IQR)	11.0 (6–30)	9.0 (6–16)	9.0 (3.5–17.5)	16.0 (6–48)	0.025
Complete small bowel visualization	122 (91.7)	21 (100)	33 (94.3)	68 (88.3)	0.247
Follow up duration in month, median (IQR)	26.0 (14.1–48.7)	34.5 (21.5–44.6)	24.3 (13.3–46.1)	26.0 (14.1–48.7)	0.580

Data are presented as number (percentage) unless indicated otherwise

*CE* capsule endoscopy, *SD* standard deviation, *GI* gastrointestinal, *HBV* hepatitis B virus, *HCV* hepatitis C virus, *NSAIDs* non-steroidal anti-inflammatory drugs, *Hb* hemoglobin, *IQR* interquartile range

### CE findings and interventions

Out of the 133 patients, 56 (42.1%) were CE positive (P2 lesion). Nine patients had P1 lesion and 68 patients had P0 lesions. The P2 lesions were identified as small bowel ulcers (20), angiodysplasia (17), small bowel tumors (10) and abnormal vessels (9).

In 35 (62.5%) patients with positive CE, specific treatments were performed, which included therapeutic DAE, embolization, surgical treatment and medication treatment (Table 2). DAE with hemostatic treatments; with either argon-plasma coagulation or electrocoagulation; were performed in 14 patients with angiodysplasia. Hemoclips were applied for abnormal vessels and Dieulafoy’s lesions in two patients. Surgical treatments, with or without intraoperative enteroscopy, were performed in eight patients with small bowel tumor, for example, gastrointestinal stromal tumor, adenocarcinoma of the small intestine and in two patients with arteriovenous malformation. An angiogram with embolization was performed on two patients with abnormal vessels. Seven patients received medication treatment. Five patients with NSAID-associated small bowel ulcers received rebamipide. Thalidomide was used as the anti-angiogenic agent in one patient with angiodysplasia, who was suspected as having hereditary hemorrhagic telangiectasia, and one patient with diffused small bowel ulcers, being diagnosed as Crohn’s disease, received prednisolone and azathioprine.

**Table 2:** Specific treatments performed for each type of lesion in patients with positive CE

Treatment	Type of lesion	Number of patients
Therapeutic device-assisted enteroscopy		16
- Argon-plasma coagulation, electrocoagulation	Angiodysplasia	14
- Hemoclips	Abnormal vessels and Dieulafoy’s lesion	2
Surgical treatment		10
	Small bowel tumor	8
	Arteriovenous malformation	2
Embolization	Active bleeding abnormal vessels	2
Medication treatment		7
- Muco-protective agent (rebamipide)	NSAID-associated small bowel ulcer	5
- Anti-angiogenic agent (thalidomide)	Hereditary hemorrhagic telangiectasia	1
- Prednisolone and azathioprine	Crohn’s disease	1

*CE* capsule endoscopy, *NSAIDs* non-steroidal anti-inflammatory drugs

Twenty-one patients with positive CE, who did not receive specific treatments, included 14 patients with small bowel ulcers without active bleeding, three patients with inactive abnormal vessels and two patients with angiodysplasia. Those patients decided to wait and see during follow-up. However, two additional patients with small intestinal tumors (gastrointestinal stromal tumor of the jejunum and appendix tumor) refused to receive aggressive surgical treatment, due to old age and the high risk of surgery.

In patients with negative CE, DAE and embolization were performed in four patients, because of active bleeding and an unstable hemodynamic status. Other patients with negative CE and inactive bleeding received conservative treatment.

This study showed the diagnostic yield of CE was higher in patients with overt OGIB than those with occult OGIB (50.6% versus 28.8%, *P* = 0.02).

### Long-term outcome in re-bleeding

**Fig. 2:**
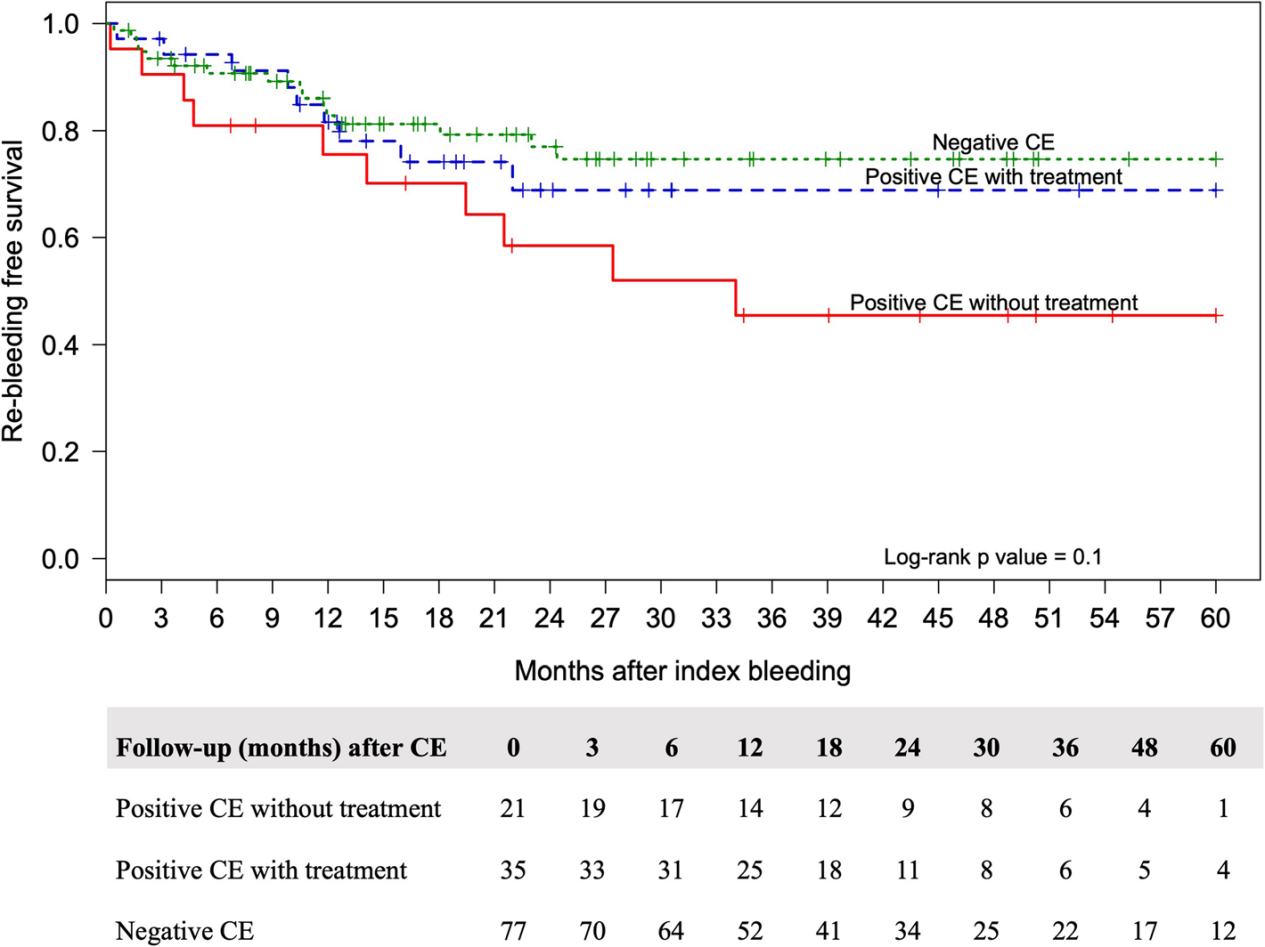
Kaplan-Meier curves show re-bleeding free survival and number of patients at risk in each group

The overall median time of follow-up was 26 months (range 14–48.7 months). Of the 133 patients, 35 patients (26.3%) showed re-bleeding events during follow-up. Patients with positive CE study without specific treatment had a significantly higher re-bleeding rate, (47.6%) than patients with positive study whom received specific treatments (25.7%), and negative study (20.8%), respectively (*p* = 0.042; log rank test) (Fig. 2). In the group of 35 patients with re-bleeding events, 21 (60%) patients presented with overt bleeding and 14 (40%) patients had occult bleeding. Additionally, the re-bleeding events after CE that occurred within 6, 12 and 24 months were 36, 64 and 92%, respectively.

EGD, colonoscopy, DAE, computer tomography with angiography, repeat CE, or surgical treatment were performed after the re-bleeding events, so as to identify the etiologies of recurrent bleeding after the initial CE.

Among 56 patients with positive findings on the initial CE, re-bleeding occurred in 19 (34%) patients of which 6 cases were angiodysplasia. The other cases were 4 vascular lesions (aneurysm or Dieulafoy’s lesion), 3 ulcerative lesions, 2 tumors (cancer of the appendix and gastrointestinal stromal tumor); both of whom did not receive treatment and 4 cases having unidentifiable lesions.

In the group of patients with negative initial CE results, re-bleeding occurred in 16 patients: 4 vascular lesions (aneurysm, Dieulafoy’s lesion), 2 small bowel diverticular bleeding, 2 angiodysplasia, 2 ulcers and 6 lesions which were not identifiable. The highest rate of re-bleeding was seen in patients with abnormal vessels (44%) and angiodysplasia (35%), whereas a lower rate of re-bleeding was observed in patients with ulcers (15%).

### Predictor analysis of re-bleeding

**Fig. 3:**
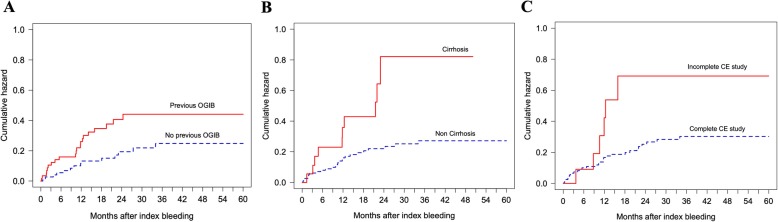
Cumulative incidence of re-bleeding and associated predictive factors. **a**. Previous overt GI bleeding before CE, **b**. Cirrhosis, **c**. Incomplete small bowel visualization

The predictors for re-bleeding after CE were analyzed using univariate analysis; wherein, the Cox proportional hazard model revealed that: a history of previous overt GI bleeding prior to CE study, hemoglobin (Hb) level < 7.0 g/dL at initial bleeding, liver cirrhosis as well as incomplete small bowel visualization of CE study were associated with re-bleeding episodes. Multivariate analysis revealed that only patients with cirrhosis (hazard ratio [HR] 4.06; 95% CI 1.88–8.78; *P* <  0.001), incomplete CE visualization (HR 2.97; 95% CI 1.23–7.66; *P* = 0.046) and a history of previous GI bleeding (HR 2.80; 95% CI 1.40–5.58; *P* = 0.003) were the significantly independent predictors for re-bleeding (Table 3). Cumulative incidence of re-bleeding coupled with significantly associated predictive factors is displayed in Fig. 3.

**Table 3:** Univariate analysis and multivariate analysis of the predictors for re-bleeding after CE

Variable factors	OGIB with CE (*N* = 133)					
	No re-bleeding (*n* = 98)	Re-bleeding (*n* = 35)	Univariate *p*-value	Multivariate† *p*-value	Hazard ratio	95% CI
Gender (Male)	46 (46.9)	18 (51.4)	0.795	–	–	–
Age ≥ 70 years	44 (44.9)	20 (57.1)	0.295	–	–	–
Overt GI bleeding (melena or hematochezia)	58 (59.2)	23 (65.7)	0.633	–	–	–
Hemodynamic instability	9 (9.2)	7 (20.0)	0.065	0.411	1.47	0.59–3.71
Hb level < 7.0 (g/dL), mean (SD)	7.5 (1.9)	6.8 (2.0)	0.075	0.498	0.78	0.38–1.60
Drop in Hb level (g/dL), mean (SD)	3.7 (1.8)	3.7 (1.8)	0.797	–	–	–
Blood transfusion ≥3 units	41 (41.8)	16 (45.7)	0.842	–	–	–
Previous overt GI bleeding	37 (37.8)	21 (60.0)	0.038	0.003	2.80	1.40–5.58
Ischemic heart disease	23 (23.5)	12 (34.3)	0.101	0.071	1.98	0.97–4.06
Aortic stenosis	4 (4.1)	2 (5.7)	0.653	–	–	–
Hypertension	47 (48.0)	18 (51.4)	0.876	–	–	–
Diabetes mellitus	28 (28.6)	10 (28.6)	0.841	–	–	–
Cirrhosis	8 (8.2)	10 (28.6)	0.007	< 0.001	4.06	1.88–8.78
Chronic hepatitis (HBV, HCV)	6 (6.1)	2 (5.7)	0.783	–	–	–
Chronic kidney disease	18 (18.4)	8 (22.9)	0.744	–	–	–
Thrombocytopenia	2 (2.0)	6 (17.1)	0.004	0.525	1.44	0.41–5.64
Continued aspirin use	11 (11.2)	5 (14.3)	0.762	–	–	–
Continued dual antiplatelet use	10 (10.2)	3 (8.6)	0.702	–	–	–
Continued anticoagulant use	4 (4.1)	2 (5.7)	0.653	–	–	–
Continued NSAID use	3 (3.1)	1 (2.9)	0.782	–	–	–
Incomplete small bowel visualization	5 (5.1)	6 (17.1)	0.037	0.046	2.97	1.23–7.66

Data are presented as *n* (%) unless indicated otherwise

†Variables with a *P* value less than 0.2 were included for the multivariate analysis, using the Cox proportional hazard regression model

Abbreviations: *CE* capsule endoscopy, *CI* confidence interval, *GI* gastrointestinal, *HBV* hepatitis B virus, *HCV* hepatitis C virus, *NSAIDs* non-steroidal anti-inflammatory drugs

### Subgroup analysis of patients with negative CE findings

In 77 patients with negative study of CE, 16 (20.8%) had re-bleeding. Univariate analysis showed that previous GI bleeding, Hb level < 7.0 g/dL at initial presentation, and incomplete small bowel visualization were associated with re-bleeding. Multivariate analysis revealed that only incomplete CE visualization (HR 7.22; 95% CI 2.2–23.7; *P* = 0.001) and previous GI bleeding prior to CE study (HR 3.35; 95% CI 1.26–10.0; *P* = 0.016) were significant predictors of recurrent bleeding after CE in patients with negative study.

## Discussion

Capsule endoscopy is considered to be the first step in the investigation of OGIB, as recommended by a recent guidelines [5]. A significant finding on CE leads to specific treatment. However, CE may not detect a bleeding lesion. Furthermore, prediction of subsequent re-bleeding after CE is still inconsistent among previous eastern studies [11, 15, 17–21]. Moreover, most of the previous studies reported short-term follow-up results.

The overall diagnostic yield for CE for OGIB, in this study, was 43%; which was comparable to previous studies [8, 11, 15, 17, 21, 22]. We found the diagnostic yield of CE was higher in the overt OGIB group than in the occult OGIB group. Similar to previous data from eastern countries [7, 8, 11, 20, 21], our study found that the most frequent CE finding was small bowel ulcer (35.7%), whereas angiodysplasia, which is the most common CE finding among western populations, was found in fewer patients (30%) in our study. We also found that time to perform the CE after onset of bleeding was delayed more in patients with negative CE results (9 versus 16 days, *P* = 0.025). Our results supposed that performing CE early, after bleeding, increased the diagnostic yield for CE, which was also confirmed by recent studies [23, 24].

Although specific treatments could have been performed in patients with angiodysplasia or abnormal vascular lesions, which are considered to be high risk re-bleeding lesions, 37.5% of these patients did not receive specific treatments in our study. The reasons included (I) bleeding spontaneously stopped, (II) some patients refused to receive invasive treatments, such as therapeutic DAE or surgery; due to their advanced age, and (III) the high risk for an invasive procedure. Some patients chose conservative treatment and follow-up rather than receiving immediate, invasive treatment after CE.

This study revealed that re-bleeding was significantly greater in patients with a positive study without specific therapy than those with positive study undergoing specific therapy and negative study, respectively; over a long period of follow-up. Our results differed from earlier eastern studies which claimed that re-bleeding in patients with positive and negative CE did not differ. Endo et al. [16] found a significantly higher rate of re-bleeding in patients with a negative CE compared to patients with positive findings (50% versus 26.7%). Park et al. [8] reported comparable re-bleeding rates of 34.8 and 35.7% from positive and negative CE exams, respectively. In a previous retrospective study [20], the re-bleeding rate was higher after a negative CE study, compared with that of a positive examination (18% versus 5.4%, *P* = 0.08). However, these studies had relatively short follow-up durations, lower diagnostic yield of CE and a lower rate of complete small bowel visualization.

Our results were consistent with a study by Lai et al. [19]^,^ which claimed that negative CE correlates with a low re-bleeding rate. A study from China, with a larger sample size that enrolled 339 OGIB patients with a follow-up period of 4 years, this study concluded that: significantly higher re-bleeding events in patients with a positive CE, as compared to patients with a negative CE (36.5% versus 13.7%, *P* = 0.0001). Moreover, a recent meta-analysis by Yung et al. [25] concluded that pooled re-bleeding rates were higher in positive CE compared to negative CE patients (29% versus 19%, *P* <  0.0001).

The re-bleeding-free survival tended to be higher in patients with negative CE than in patients with positive CE, who received specific treatments and those with positive CE, who did not receive any specific treatment. However, there was no significant difference among the groups (*P* = 0.05, log-rank test). We suspect it resulted from the small number of patients who had re-bleeding after 24 months of follow-up.

Our findings found that the percentage of re-bleeding was lower in patients who received specific treatment, this was consistent with a previous study by Tan et al. [13] (22.4% versus 34.9%, *P* = 0.007). On the contrary, some studies reported that the re-bleeding rate did not differ between patients with or without specific treatments [7, 18, 26, 27].

Re-bleeding events in OGIB patients after CE occurred in 64 and 92% within the follow-up periods of 12 and 24 months, respectively. This result was consistent with a previous study by Tan et al. [13] that indicated 95.9% experienced re-bleeding events within 24 months after CE. Therefore, long-term close observation for at least 2 years after CE should be considered, even though specific treatments for significant lesions were given.

After an initial positive CE study, small bowel angiodysplasias and vascular abnormalities were found to be the high frequent lesions for re-bleed. These lesions tend to recur and re-bleed in their natural course, whereas small bowel ulcers, which are the common culprit lesions, perhaps spontaneously heal after withdrawal of NSAIDs with a low potential to re-bleed.

This present study demonstrated that patients with cirrhosis, incomplete small bowel visualization and one or more episodes of GI bleeding, prior to CE examination, were the independent predictors for re-bleeding. These risk factors were also reported by previous studies. Pennazio et al. [22] reported that previous overt GI bleeding was the risk factor for re-bleeding. Niikara et al. [21] reported cirrhosis was the risk for re-bleeding after CE. In this study, incomplete small bowel visualization was found as a strong, new predictive factor in patients with either positive or negative CE. This suggests that further investigations or repeated CE examination should be considered in cases of incomplete small bowel visualization.

Since previous overt bleeding was a strong predictive factor in this present study, our hypothesis is that the natural history of the lesions, such as angiodysplasia and aneurysm, had a high potential to recur and re-bleed. Cirrhosis was found to be a risk factor for recurrent bleeding from portal hypertensive enteropathy or hemostatic disorders. In addition, incomplete small bowel visualization was also an unquestionable predictive factor for re-bleeding, because the lesions may go undetected and untreated.

The strengths of this study included the long follow-up time (median 26 months) and the predictive factors being evaluated in multivariate analyses. We also provided data classified by CE results and specific treatments and a subgroup analysis for negative CE patients. Our study also reported that the rate of complete small bowel visualization was 92%, which was higher than 64 to 80% reported in previous studies.

This study has some limitations. First, the study is of a retrospective design. Second, a relatively small sample size was not able to demonstrate a significant difference in survival analysis. A large number of OGIB patients were excluded from the analysis, due to insufficient data assessment of re-bleeding events on follow-up visits. In addition, the evaluation of onset for re-bleeding in patients with occult re-bleeding was not accurate, because its presentation was likely to be asymptomatic. Patients with iron deficiency anemia who received iron supplement may maintain their hemoglobin level and conceal minor re-bleeding events.

## Conclusions

Re-bleeding rates after CE in OGIB was higher in patients with a positive CE study, when compared to patients with a negative CE study in long-term follow-up. Additionally, specific treatments for patients with detectable lesion on CE reduced the probability of re-bleeding episodes. The independent predictors associated with re-bleeding were; cirrhosis, incomplete small bowel visualization and a history of previous GI bleeding. For the above reasons, further diagnostic, or treatment modalities should be considered for patients with predictive factors of re-bleed. Hence, close follow-up for at least 2 years after CE is recommended.

## Data Availability

The datasets used and/or analyzed during the current study are available from the corresponding authors upon reasonable request.

## Abbreviations

CE: Capsule endoscopy
DAE: Device-assisted enteroscopy
EGD: Esophagogastroduodenoscopy
GI: Gastrointestinal
Hb: Hemoglobin
NSAIDs: Non-steroidal anti-inflammatory drugs
OGIB: Obscure gastrointestinal bleeding
